# Population receptive field tuning properties of visual cortex during childhood

**DOI:** 10.1016/j.dcn.2019.01.001

**Published:** 2019-01-08

**Authors:** T.M. Dekker, D.S. Schwarzkopf, B. de Haas, M. Nardini, M.I. Sereno

**Affiliations:** a Institute of Ophthalmology, University College London, UK; b Experimental Psychology, University College London, UK; c School of Optometry & Vision Science, University of Auckland, New Zealand; d Department of Psychology, Justus-Liebig-Universitat, Giessen, Germany; e Department of Psychology, Durham University, UK; f Dept. of Psychology, San Diego State University, USA

**Keywords:** pRF mapping, Vision, Retinotopy, fMRI, Childhood

## Abstract

Visuospatial abilities such as contrast sensitivity and Vernier acuity improve until late in childhood, but the neural mechanisms supporting these changes are poorly understood. We tested to which extent this development might reflect improved spatial sensitivity of neuronal populations in visual cortex. To do this, we measured BOLD-responses in areas V1-V4 and V3a, whilst 6- to 12-year-old children and adults watched large-field wedge and ring stimuli in the MRI scanner, and then fitted population receptive field (pRF) tuning functions to these data (Dumoulin and Wandell, 2008). Cortical magnification and pRF tuning width changed with eccentricity at all ages, as expected. However, there were no significant age differences in pRF size, shape, cortical magnification, or map consistency in any visual region. These findings thus strongly suggest that spatial vision in late childhood is not substantially limited by the spatial tuning of neuronal populations in early visual cortex. Instead, improvements in performance may reflect more efficient read-out of spatial information in early visual regions by higher-level processing stages, or prolonged tuning to more complex visual properties such as orientation. Importantly, this in-depth characterisation of the pRF tuning profiles across childhood, paves the way for in-vivo-testing of atypical visual cortex development and plasticity.

## 1 Introduction

Building a visual system with adult-like capabilities involves extensive shaping of neural mechanisms through experience (Braddick and Atkinson, 2011; Hubel and Wiesel, 1962). The greatest changes in vision occur in the first year of life, but many visuospatial skills improve throughout the first decade. For example, contrast sensitivity matures between the ages of 7–9 years for gratings (Adams and Courage, 2002; Benedek et al., 2003; Ellemberg et al., 1999; Leat et al., 2009) and at ∼age 10 for naturalistic textures (Ellemberg et al., 2012, 2009), whilst Vernier acuity (positional resolution) only converges on adult performance between 10–14 years (Carkeet et al., 1997; Skoczenski and Norcia, 2002). Larger-scale spatial integration, such as acuity for shapes surrounded by flankers (crowding), size illusion sensitivity, contour integration, face perception, and object perception, also have been found to develop into the teenage years (Bova et al., 2007; Doherty et al., 2010; Golarai et al., 2007; Hadad et al., 2010; Hipp et al., 2014; Jeon et al., 2010; Kovács, 2000). Understanding what drives these substantial late changes is important for determining the types of plasticity we may expect at different ages across childhood and after. This is becoming an increasingly important question with the emergence of novel treatments for eye disease that restore retinal function, and are likely to work best at earlier ages (Carvalho et al., 2011). We therefore used population receptive field (pRF) mapping (Dumoulin and Wandell, 2008) to investigate whether late improvements in spatial vision in childhood are reflected in prolonged spatial tuning of neuronal populations across the visual hierarchy.

Receptive fields of neurons in visual cortex maintain a retinotopic organisation, with adjacent neurons encoding adjacent areas of the visual field. There are multiple copies of the visual field along the cortical hierarchy (V1, V2, V3, etc.) (Wandell et al., 2007). In accordance with the disproportionate degree of cortical input from the fovea, where receptor density is highest, more visual cortex surface is dedicated to the visual field near the fovea. The visual distance encoded per mm cortex (cortical magnification), decreases with viewing eccentricity (Sereno et al., 1995). Visual acuity is correspondingly greatest in foveal parts of the field. In addition, neurons encoding smaller eccentricities typically have smaller receptive fields than those encoding the periphery, which gives them greater spatial resolution. Population receptive field (pRF) mapping fMRI was recently developed to measure such visuospatial tuning in the living human brain (Dumoulin and Wandell, 2008). To obtain these measures, different parts of the visual field are stimulated succesively and systemetically, using a screen viewed in an MRI scanner. The visual field locations at which these stimuli evoke BOLD-responses in visual cortex are recorded. Each voxel’s BOLD-response is then fitted with the predicted response of a population receptive field model that describes the voxel’s preferred retinotopic location (the pRF position) and the area around this location that the voxel responds to (the pRF size). In its simplest form, the population receptive field is modelled as an excitatory tuning filter (a bivariate Gaussian distribution). More complex pRF models inspired by single neuron receptive fields, may also incorporate a surround suppression component around the excitatory centre (a difference of Gaussian distribution; Zuiderbaan et al., 2012). pRF tuning properties in human adults mirror those of single cell receptive fields in that towards the periphery pRF size increases in visual cortex areas encoding the periphery (tuning functions become wider) and cortical magnification decreases (less cortex encodes the same visual distance). However, because functional MRI measures brain activity as reflected in changes in blood flow at a resolution of 1-3mm^2^, it collapses across receptive fields of thousands of individual neurons and their interactions. Recent studies have shown associations between individual adults’ pRFs and their Vernier acuity (Duncan and Boynton, 2003; Song et al., 2015), and found altered pRF tuning profiles in several populations with altered visuospatial processing (Anderson et al., 2017; Brewer and Barton, 2014; Clavagnier et al., 2015; Schwarzkopf et al., 2014). Thus, pRFs in early visual cortex appear highly relevant for visuospatial skills.

Little is known about visuospatial resolution in the developing human brain. Histological studies suggest that the fovea is still not mature by 45 months of age (∼4 years) (Yuodelis and Hendrickson, 1986), and occipital cortex continues to thin and expand until ∼age 10 years (Ducharme et al., 2016; Jernigan et al., 2016), there is substantial myelination of visual cortex, and cortico-cortical connectivity between V1 and higher order brain areas increases substantially. However, the changes in neural functioning that accompany these structural changes along the visual pathways are unknown. Here we investigate whether these changes are paired with changes in visuospatial profiles in visual cortex, by measuring pRFs across visual cortex in 6 to 12-year-old children and adults.

The relationships between population receptive fields in visual cortex, classic receptive fields, and visual perception is still poorly understood and not always intuitive (Kay et al., 2015; Smittenaar et al., 2016). However, we formulated several speculative hypotheses for how age-related improvements in visual perception between ages 6–12 years might be reflected in changes in pRF tuning. Firstly, we expected that improvements in Vernier acuity, contrast sensitivity, and foveal crowding reported at the age of 6 years and beyond, might be paired with changes in pRF shape and size (hypotheses i&ii). Specifically, we hypothesised that (i) *pRFs might be larger in early childhood compared to adulthood*, since coarser spatial tuning functions could reduce the ability to resolve fine detail, and enhance crowding through suppression from surrounding stimuli across larger distances. We also hypothesised that (ii) *children might show greater surround suppression than adults*, as greater crowding has been linked to stronger cortical inhibition (Chen et al., 2014; Millin et al., 2014). In addition, we expected that these age-related improvements in visual perception might be paired with changes in the distribution of pRFs across the cortical sheet (hypotheses iii&iv). Specifically, we expected *(iii) age-related increases in cortical magnification*, since greater cortical magnification also correlates with higher Vernier acuity in adults (Duncan and Boynton, 2003). Finally, we tested for *(iv) increases in the consistency of retinotopic layout*, reasoning that this would capture age-related reductions in pRF position scatter that may emerge with prolonged refinement of the retinotopic map, and which may yield greater precision of spatial information (Clavagnier et al., 2015; Haak et al., 2013). Alternatively, if pRF properties become adult-like early, late improvements in visuospatial performance may reflect more efficient information read out by higher-level neural mechanisms, independent of spatial tuning in early visual cortex. Distinguishing between these possibilities, and characterising how pRF properties change across childhood is important for establishing a typical development benchmark of visual cortex function, against which children with impaired vision can be compared.

## 2 Methods

### 2.1 Subjects

We tested 39 children aged 6–12 years and 7 adults: all with normal or corrected to normal vision, and no known neurological abnormalities. To ensure that any age-differences in BOLD response-dependent measures would not be driven by movement-related noise, we used stringent exclusion criteria; participants who made large movements in the scanner were excluded (a 1 mm translation, or 3° rotation during more than 3 volumes collected across all functional scans; see Section 3. Data Quality Assurance and Supplementary Fig. 1). This cut-off resulted in matched movement parameters across all age groups. The remaining participants included in the analysis were thirteen 6–9-year-old children (8.23 years, SD = 0.89), seventeen 10- to 12-year-old children (Mean Age: 11.39, SD = 0.74), and seven adults (22.30 years, SD = 2.72). All participants had normal or corrected to normal vision, no known neurological abnormalities, met MR-safety criteria, and provided written informed (and in parental) consent. Experimental procedures were approved by the UCL Research Ethics Committee

### 2.2 Scanning parameters

Structural and functional measures were obtained with a Siemens Avanto 1.5 T MRI scanner and 30-channel coil (a customized 32 channel coil without obstructed view). BOLD measures were acquired using four single-shot EPI runs (TR = 2.5 s, volumes = 144, slices = 30 voxel size = 3.2 × 3.2 x 3.2 mm, axial plane, ascending, bandwidth = 1930 Hz/pix, TE = 39 ms, flip = 90). The high-resolution structural scan was acquired using a T1-weighted 3D MPRAGE (1 mm^3^ voxel size, Bandwidth = 190 Hz/pix, 176 partitions, partition TR = 2730, TR = 8.4 ms, TE = 3.57, effective TI = 1000 ms, flip angle = 7 °).

### 2.3 Stimuli

pRF mapping stimuli were back-projected on an in-bore screen at 34 cm viewing distance (projected area: 18 x 24 cm; resolution: 1920 × 1080). They consisted of moving eccentricity-scaled ring and wedge checkerboards presented against a dark grey background. During odd runs, the ring expanded for 10 cycles (32 s/cycle), and the wedge (21° angle) rotated anticlockwise for 6 cycles (53.33 s/cycle). We chose a wedge and ring stimulus because this configuration yielded the the most reliable fit in a direct comparison of different pRF mapping stimulus configurations (Alvarez et al., 2015). During even runs, the ring contracted and the wedge rotated clockwise at the same speeds. The stimuli covered a maximum vertical eccentricity of 14.8°, and moved to a new position each new TR. They were overlaid with a white central fixation dot (0.3 ° radius) and a white radial grid to anchor fixation. Checkerboards had a fixed contrast (35%), achieved by randomly selecting hue with constant saturation and two levels of lightness. Contrast reversals occurred at 8 Hz. Checkerboards were superimposed with moving dots (diameter: 0.08°; randomly rotating and expanding or contracting at speeds between 0°-19°/sec) and briefly presented photos of animals and household items (0.6 s/image). Movement, color, and objects were added to elicit maximal retinotopic responses across visually driven cortex, and make the stimuli more appealing to children. The ring and wedge were preceded and followed by a 20-second (8 TR) baseline.

### 2.4 Procedure

Participants “practiced” being scanned and lying still whilst watching a funny 5-minute cartoon and undergoing a short localizer scan. Then, participants completed 4 functional runs of 6 min with two “cartoon breaks” in-between when structural data were collected. To keep participants engaged and motivated to fixate during pRF mapping, they could score points by detecting changes in the fixation target via a button-press. These changes involved a brief brightening of the target or a letter superimposed on it, each occurring probabilistically at 0.2/sec. Scores (% detected targets) were shown at the end of each run. Children were carefully monitored via an in-bore face camera and an intercom to ensure that they kept fixating and lying still comfortably during each run, and that they were happy to continue.

### 2.5 Analysis

Freesurfer (http://surfer.nmr.mgh.harvard) was used to reconstruct the cortical surface from the structural scan, and SPM8 (http://www.fil.ion.ucl.ac.uk/spm) was used to pre-process functional data. Preprocessing involved realignment, slice-time correction, and computing a co-registration matrix. Functional data was then projected onto the reconstructed cortical surface mesh, by sampling time courses from voxels midway between the white and grey matter surface. Linear trends were removed and time courses were normalized.

We used the SamSrf Matlab toolbox for population receptive field model fitting (https://doi.org/10.6084/m9.figshare.1344765). As pRF model, we took a bivariate Gaussian distribution with free parameters X, Y, and Sigma, corresponding to the preferred retinotopic coordinate and pRF size respectively. To predict the response of each pRF model to the stimuli, we integrated the bivariate Gaussian described by each model across a binarised stimulus image for each TR. There are no substantial age differences in HRF across the tested age range (Moses et al., 2014; Richter and Richter, 2003). Therefore, the resulting time series was convolved with a standard HRF function (Haas et al., 2014) to account for delays in the BOLD response at all ages.

To identify which pRF model (which X, Y and Sigma) best predicted the measured time course, we then used a two-step procedure. (1) In a coarse fitting step, we applied heavy spatial smoothing (FWHM kernel = 8.3 mm on the spherical surface mesh) to reduce local minima. We then took a coarse, 3-dimensional search grid of the parameter space, and computed for each cortical surface vertex which parameter combination yielded the highest Pearson correlation between the measured and predicted time series. These highest-correlating parameters were used as starting point for the subsequent fine fitting step, unless R^2^ < 0.05, in which case the vertex was discarded. This is a default threshold for this pRF fitting method, and we obtained similar results with a more stringent threshold R^2^ < 0.1. (2) In a fine fitting step, we used multidimensional unconstrained nonlinear minimization as implemented in the fminsearch function in Matlab, to compute the pRF parameters that minimized the difference between the predicted time course and the unsmoothed time course data. Next to X, Y and Sigma, this step also fitted a parameter Beta, accounting for signal amplitude.

We also fitted the data with a more complex Difference of Gaussian pRF model. This model has two additional free parameters next to the X, Y, and Sigma of the simple Gaussian pRF. These include the size of a second wider Gaussian (Sigma2), which was subtracted from the original positive component, and a scaling variable that determines the ratio of the two subtracted Gaussians (DoG-ratio). These added components effectively modulated the width of the positive Gaussian kernel and added a negative surround to create a “Mexican hat” shape (Zuiderbaan et al., 2012).

**Fig. 1:**
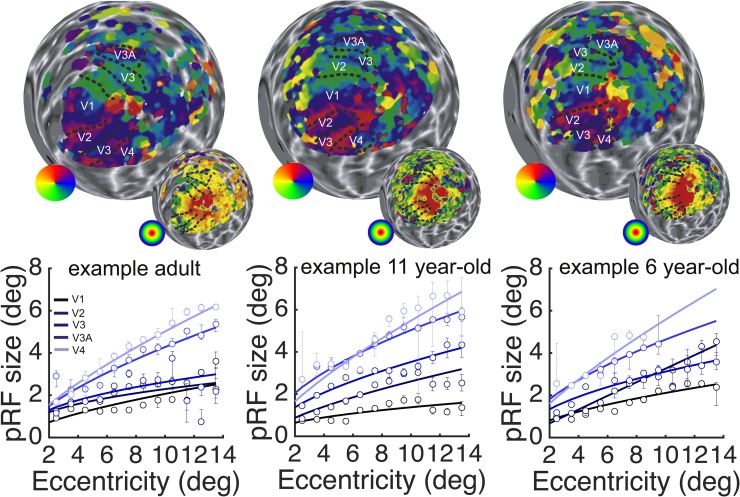
Polar angle (large) and eccentricity (small) activation maps are displayed on the reconstructed left hemisphere (inflated to sphere) for a representative example participant in each age group. Retinotopic regions of interest defined manually based on horizontal and vertical meridians on the polar angle map are delineated. Graphs display the pRF size (the σ parameter of the best-fitting Gaussian pRF model) in degrees v/a plotted against eccentricity for ROIs in the left hemisphere. Circles indicate median pRF size of all vertices within the eccentricity bin with R^2^>0.1, error bars are bootstrapped 95CIs. Data points from eccentricity bins with <10 vertices with R^2^>0.1 are omitted. Lines display the best fitting 1st order power function fitted through these data points. Different shades of blue indicate measures from different visual areas.

Only vertices in which the best-fitting pRF model had a good fit (R^2^>0.1) were included in the analyses. To compute polar angle maps we took the counter-clockwise angle between the positive X-axis and the polar X, Y coordinate of the best-fitting pRF model (atan (Y/X)). To compute eccentricity maps, we took the Euclidean distance from the polar X, Y coordinate to the origin at fixation (sqrt(X^2^+Y^2^)). For in-depth description of these methods see (Alvarez et al., 2015). We manually delineated visual regions of interest V1-V3, V4, V3A, based on horizontal and vertical meridians in the polar angle map, following standard criteria (see Fig. 1a). We also attempted to delineate higher-order areas such as MST and V6, but did not include these areas in the analysis because in many participants they could not be identified reliably and model fits were poor in these areas. Future studies may investigate development of these areas using larger field of views and/or separate localisers.

## 3 Data quality assurance

### 3.1 Head movement

We used stringent inclusion criteria to minimize any contributions from head-movement to any age differences in functional activation (see section 2.1. Subjects, for details). After excluding participants who did not meet our criteria, there were no significant age differences in translation or rotation (translation: F(233) = 0.14, p = 0.87, rotation: F(233) = 0.12, p = 0.89, see Supplementary Fig. 1).

### 3.2 Fixation task performance

Participants were observed via an in-bore camera throughout the experiment and reminded to keep fixating when necessary. All age groups detected a high proportion of central target changes during the scanning runs (7 to 9-year-olds: 0.88 (SD = 0.05); 10 to 12-year-olds: 0.87 (SD = 0.05); adults: 0.90 (SD = 0.05), with no significant age difference revealed by a bootstrapped 3-way ANOVA (p = 0.08). Thus, all participants attended well to the central marker throughout the experiment.

### 3.3 Goodness of pRF model fit

Another way of ensuring that data quality was equivalent across age groups is by comparing the goodness of fit of the population receptive field model to the data. Although median Goodness of Fit (based on the Gaussian pRF model) was slightly better in adults, this difference did not reach statistical significance (F(234) = 2.23, p = 0.12). However, after removing vertices with a poorer data quality (R^2^ < 0.1) from the data, adults had slightly but significantly better model fit (F(234) = 3.42, p = 0.04).

## 4 Results

### 4.1 Population receptive field size and shape

To test for age-related changes in population receptive (pRF) size *(hypothesis i)*, we first extracted the average pRF size from each visual area of interest. PRF size was defined as the standard deviation (Sigma) of the best-fitting symmetric bivariate Gaussian. We then binned each vertex in the ROI by its eccentricity (14 bins from 1-15°) and computed the median Sigma for each bin. Only vertices with a good pRF model fit (R^2^ > 0.1) were included. Fig. 1, shows delineated borders of V1-3, V4 and V3A and example data from these regions for three individual representatives of their age group. In line with previous reports, pRF size increases with eccentricity and across the visual hierarchy for each participant. This mirrors receptive field size profiles derived from single neuron recordings.

**Fig. 2:**
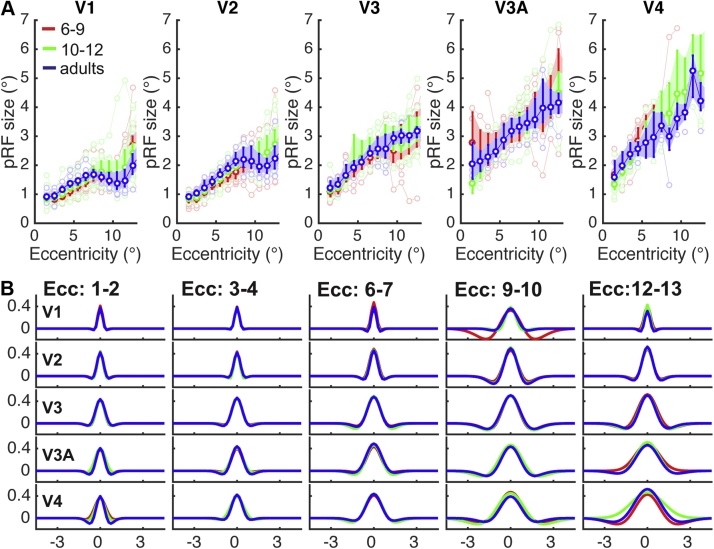
a) average pRF size (σ) per age group plotted across eccentricity. Shaded errorbars are bootstrapped 95%CIs. Individual data is plotted in faint colours. b) Average Difference of Gaussian pRF model in each age group are plotted for 4 representative eccentricity bins, x-axis depicts width in degrees visual angle, y-axis depicts height.

To compare how receptive field size changed across age, we took group average data (Fig. 2a), and used bootstrapped ANOVA’s to test for age-differences in Sigma at each eccentricity. As the overlap in bootstrapped confidence intervals across the three different age groups indicates, there were no significant age differences in pRF size in any of the regions of interest. Out of 70 tests (5 ROI x 14 eccentricity bins), the smallest p-value was 0.059 at eccentricity bin 7-8° in V2. None of the p-values were significant at a false discovery rate of 0.05 (Benjamini and Yekutieli, 2001).

Neural receptive fields with center-surround configuration responses that are modulated by adjacent inputs are abundant in the visual system. These types of spatial dynamics are not fully captured by a Gaussian model with only an excitatory component, but are better explained by a Difference of Gaussian model with a surround inhibition component that gives it a Mexican-hat shape (Zuiderbaan et al., 2012). Therefore, to test for age-related changes in pRF shape *(hypothesis ii)* that capture the excitation/surround suppression balance in visual cortex, we fitted this more complex pRF model to the time course data. In Fig. 2b, average best-fitting DoG models in V1, V2, V3, V3A, or V4 are plotted across eccentricity for each age group. As with the Gaussian pRF model, the positive kernel of the DoG pRF increases with eccentricity and along the visual hierarchy, as does the inhibitory component that models the surround modulation. Deviations from this pattern and more variability in pRF shape at larger eccentricities are likely due to interactions with receptive fields just beyond the edge of the stimulus at 14.8°, and do not indicate significant differences across age groups; Bootstrapped ANOVAs comparing the three parameters of the DoG model across age per eccentricity bin revealed no significant age differences. Out of the three DoG parameters, the smallest *p*-value was 0.005. No *p*-values were significant at a false discovery rate of 0.05 (see Supplementary Fig. 2). This suggests that there are no substantial changes in surround inhibition of pRFs in visual cortex between ages 6–12 years and adulthood.

### 4.2 Population receptive field distribution

**Fig. 3:**
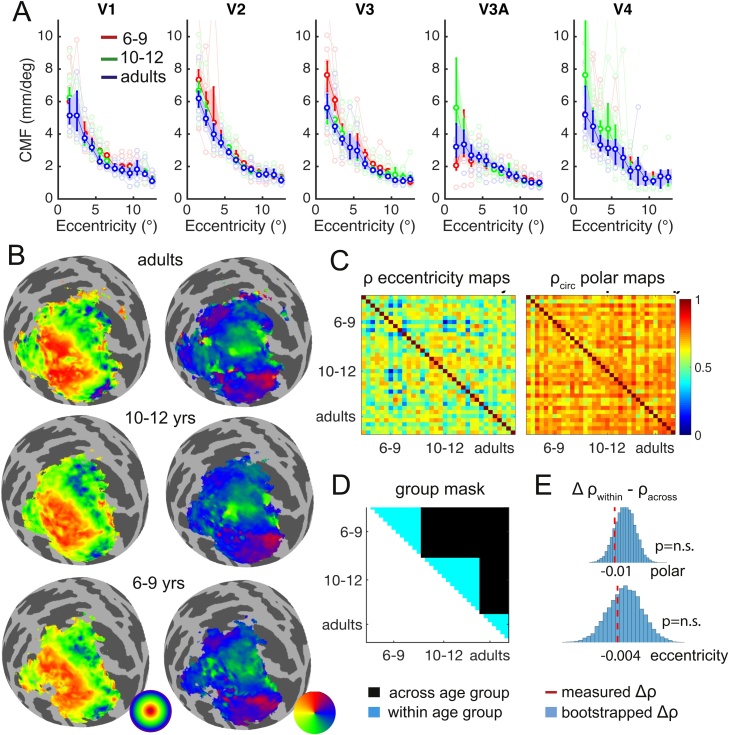
a) Cortical magnification factor across age and visual ROI. Errorbars are bootstrapped 95%CI b) Retinotopic maps averaged across participants in each group. They are projected onto the cortical surface of the occipital lobe inflated to a sphere (left hemisphere only). Eccentricity maps (left column) and polar angle maps (right column) are shown for each age group. Only vertices with R^2^ <0.05 are included in the average. c) Matrices visualizing similarity of eccentricity maps quanitified by Pearson’s correlations (left) and similarity of polar angle maps quantified by circular correlations (right) in occipital cortex across all pairs of participants, sorted by age. d) Group mask for correlation comparison. Black areas: correlations averaged to compute similarity across age groups, blue areas: correlations averaged to compute similarity within age groups. e) Red dashed lines indicate the difference in mean correlation within vs. across age groups for polar (top) and eccentricity (bottom). Negative values indicate larger correlations across groups than within. Blue H_0_ distributions were obtained by shuffling correlations randomly across individuals without respecting age group boundaries, and computing the mean across cells that originally contained across and within age group correlations (black and blue areas in d).

To test for changes in cortical magnification (*hypothesis iii*), we computed the cortical magnification factor (CMF), defined as the distance along the cortical surface required to represent a 1° distance across the visual field (Fig. 3a). Analogous to the pRF size and shape analyses, we then binned each vertex with R^2^ > 0.1 by its eccentricity between 1-15°, and computed the median CMF. Bootstrapped ANOVAs comparing age groups separately for each eccentricity revealed no significant age differences in cortical magnification; The smallest p-value out of 70 tests (5 ROI x14 bins) was p = 0.012, with no p-values significant at a false discovery rate of 0.05. In line with the similar pRF-sizes and cortical magnification factors across development in all 5 ROIs, the distribution of pRFs across the visual field (visual field coverage) was also similar across age groups (Supplementary Fig. 3).

**Fig. 4:**
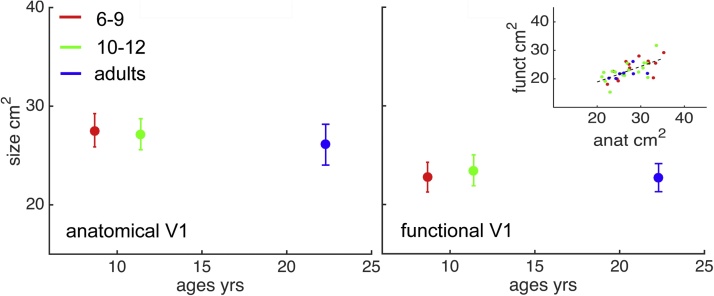
Age differences in anatomical V1 size (left) delineated using an automated algorithm (Hinds et al., 2008), and functional V1 size (right) delineated manually using retinotopic activation maps. Errorbars are 95%CI. Right inset: correlation between anatomical and functional area size. Dotted line: best-fitting linear trend.

To investigate if the overall layout of retinotopic maps changes with age, we tested for age differences in spatial consistency across individuals (*hypothesis iv*). In order to compare activation maps directly across different participants, we first aligned all individual surfaces to the Freesurfer fsaverage template so they were in a common space. We then sorted all participants by age, and calculated pair-wise Pearson’s correlations between their eccentricity maps, and pair-wise circular correlations between their polar angle maps. In Fig. 3c, we have plotted the resulting correlations across the eccentricity (left) and polar angle map (right) of each possible pair of participants in two similarity matrices. Three subjects from the 10 to 12-year-old age group were excluded from this analysis, because their cross-participant map correlation (the column or row average in Fig. 3c) was 3 mean absolute deviations from the median lower than for other participants. This most likely reflects poor alignment of their surface to the Freesurfer average due to movement artifacts in the structural scan. To ensure that activations included in this analysis covered the same cortex regions for all individuals, we excluded vertices with a poor pRF model fit (R^2^ < 0.05) in any of the participants. A more lenient cut-off (e.g., excluding vertices with R^2^ < 0.01 in more than 15 participants) yielded similar results (Fig. 4).

We tested if map similarity changed with age, by taking the average correlation between retinotopic maps within age groups (blue areas in Fig. 3d), and comparing it to the average correlation between maps across age groups (black areas in Fig. 3d). If the spatial consistency of retinotopic maps changes across the tested age range, we should expect correlations amongst participants from the same age group to be higher than amongst those of different age groups (blue cells in Fig. 3d should be “hotter” in color). This pattern is not clearly apparent when inspecting the similarity matrices in 3c, as correlations appear homogenous across all cells. Indeed, our analyses revealed a slightly higher mean correlation between individuals from *different* age groups than between individuals from the same age (Δρ_within vs. across age_ polar map = -0.01; Δρ_within vs. across age_ eccentricity map = -0.04, dotted lines Fig. 3e). This difference in the unexpected direction was not significant; it did not exceed the 95CI of a bootstrapped null-distribution that was obtained by shuffling correlations randomly across individuals and age groups (blue bars, Fig. 3e). In sum, we found no evidence for a systematic change in the distribution of population receptive tuning functions along visual cortex. Instead, cortical magnification factor and consistency of retinotopic maps were similar across children and adults.

### 4.3 V1 size

Population receptive fields and cortical magnification factor are both correlated with V1 size (Harvey and Dumoulin, 2011), and visual cortex size might still increase across the tested age range (Jernigan et al., 2016). We therefore tested if any age differences in population receptive fields might be masked by systematic differences in the cortical surface area of V1 across age groups. Fig. 1 shows anatomically- (left) and functionally- (right) defined V1 sizes per age group. Anatomical V1 borders were defined based on anatomical landmarks using Hinds’ probabilistic atlas (Hinds et al., 2008), as implemented in Freesurfer. Areas that belonged to V1 with an 80% probability were included in the structural label. Functional V1 borders were delineated manually based on the individual polar and eccentricity maps (Tootell et al., 1998; see Fig. 2). There were no significant age-differences in V1 size in either measure, so size differences are unlikely to have masked substantial development of population receptive fields. This is in line with findings from large-scale population studies showing that changes in occipital cortex across the tested age range are very small, in the order of ˜3% (Jernigan et al., 2016). Functionally defined V1 covered a smaller area of cortex than the structurally defined V1 due to limited visual field stimulation. In line with previous findings (Dougherty et al., 2003), there was substantial (˜2-fold) variability in V1 size across individuals of all ages (Fig. 1, scatterplot inset; Pearson’s correlation = 0.7, *p* < 0.001.

## 5 Discussion

A large number of studies have reported substantial improvements in basic visuospatial discrimination until late in childhood. Abilities that have been reported to improve until beyond the age of 6 years include contrast sensitivity, Vernier acuity, crowded acuity, long-range spatial integration, and face and object perception. We used population receptive field mapping to investigate to which extent this development is reflected in spatial tuning of neuronal populations across the visual cortex hierarchy. To this end, we delineated visual areas V1, V2, V3, V4, and V3A in groups of 6 to 9-year-olds, 10 to 12-year-olds, and adults, and compared population receptive field tuning curves and distributions within those areas. We used stringent head-movement exclusion criteria to ensure age-differences could not be explained by this confound.

To test for age differences in pRF size and shape, we fitted BOLD measures with a simple Gaussian model that varied freely in size and location, and with a difference of Gaussian model with a positive kernel and surround-suppression. For both models, pRF size increased with eccentricity and along the visual hierarchy in all participants, mirroring previous findings with adults using checkerboard stimuli (Dumoulin and Wandell, 2008). Moreover, we found no age differences in pRF size and surround suppression in the tested eccentricity range (up to ˜15°). We also investigated differences in the distribution of pRFs along the visual field; Cortical magnification decreased from central to peripheral eccentricities in all participants, with no systematic age-differences anywhere along the visual field. Visual field coverage for the tested cortex remained consistent across ages, and pairwise correlations of polar and eccentricity maps revealed that retinotopic organization variability was similar regardless of age group. In sum, our data revealed no substantial developmental changes in pRF size, shape, cortical magnification, visual field coverage, and consistency of retinotopic maps from the ages of 6–9 years onwards in V1-4 and V3A. Thus, pRF tuning properties of neural populations in visual cortex were close to adult-like in late childhood, despite substantial age-related improvements reported in spatial vision between the age of 6 years and adulthood (Carkeet et al., 1997; Jeon et al., 2010; Skoczenski and Norcia, 2002).

This finding is striking in the context of previous studies that have linked reduced or altered visuospatial abilities in amblyopia, old age, schizophrenia, autism, and normal adult vision, to pRF properties in early visual cortex (Anderson et al., 2017; Brewer and Barton, 2014; Clavagnier et al., 2015; Schwarzkopf et al., 2014). This discrepancy with the current study is unlikely to be due to lower power; Firstly, because our study contained similar or larger sample sizes as these previous studies. Secondly, our data met stringent quality criteria that were matched across age groups (see section 3. Data Quality Assurance). Finally, whilst it is difficult to compare perceptual thresholds across different studies, the age-related differences in visual thresholds reported in developmental literature are in principle sufficiently large to be reflected in our pRF measures. This is because differences across age groups are often substantial compared to differences within these age groups, whilst adult-sized variation in visual thresholds is related to substantial variation in pRF size and positioning. For example, by age 6 years, Vernier acuity is still about half the adult value (Carkeet et al., 1997; Jeon et al., 2010; Skoczenski and Norcia, 2002), and individual differences in adult Vernier acuity have been linked to a 300% decrease in pRF size (Song et al., 2015). In contrast, the current data constrain the plausible (95%CI) range for a pRF size difference between ages 6 to 9 years and adulthood, to between 0–30% (Supplementary Table 1). Following this line of reasoning, the current data suggest that the processes limiting the development of spatial vision in late childhood, may be different from those limiting adult vision – even though subtle age differences in pRF tuning around age 6 years may be uncovered with larger sample sizes, different methods, and better pRF model fit across child- and adulthood.

The conclusion that receptive field development in early visual cortex is not the main limiting factor on childhood spatial vision, is in line with electrophysiological recordings from young macaques; Kiorpes and Movshon (2003) reported that receptive fields encoding the central 5° of the visual field became ∼3x smaller after birth, but behavioural discrimination improved for longer and at greater rates (Jacobs and Blakemore, 1988; Kiorpes, 2015; Kiorpes and Movshon, 2003). Our study replicates the finding that pRF size develops before perceptual abilities become adult-like, and extend it in important ways by demonstrating that pRF tuning profiles in higher order areas V3, V4 and V3A are also adult-like before many visual abilities fully develop in childhood.

What can explain this discrepancy between the development of population receptive field properties and visuospatial discrimination abilities across childhood? One possibility is that development of basic visual functions such as contrast sensitivity and Vernier acuity in humans crucially depends on other neuronal tuning properties such as orientation and spatial frequency tuning. To test how these features develop across the visual field, pRF mapping and estimation methods must be expanded to more complex stimulus dimensions (Welbourne et al., 2018), or pRF shapes (Merkel et al., 2018; Silson et al., 2018).

Another possibility is that the improvements in visual perception observed in childhood requires more efficient interactions between pRFs in low-level and higher-order areas in the cortex (Kiorpes, 2015). This could for example enable better read-out of information, or more efficient task-related modulation of incoming inputs. By measuring correlations between pRF responses in V1-3 and higher-order regions in frontal, parietal, and inferotemporal cortex, such interactions may be investigated. We were unable to fit reliable pRF models in higher-order regions than the ones reported in the study, so this was not possible with the current dataset. However, in a recent study, (Gomez et al., 2018) reported that pRFs in the smaller child fusiform face area (FFA) were more clustered around the centre of gaze, and unlike the larger adult FFA, may therefore not encode entire faces seen at typical viewing distance. Importantly, in convergence with our results, these authors also found that child pRFs were similar in size to adult pRFs in earlier areas, although their analysis was restricted to a much more centrally limited part of the visual field. Since our stimuli included complex features such as hue, motion, and objects, on top of the traditional checkerboard wedge and ring, one might expect that pRF properties (e.g., size) in low-level visual cortex might be influenced by feedback from higher areas involved in processing these features. However, in line with a previous study comparing natural images versus checkerboard stimuli (van Dijk et al., 2016), the pRF properties measured in V1-3 using our complex stimuli are in high quantitative agreement with results from traditional pRF-mapping stimuli for all age groups (e.g., Alvarez et al., 2015; Dumoulin and Wandell, 2008; Schwarzkopf et al., 2014; van Dijk et al., 2016). Therefore, any effects via feedback from higher-order cortex regions on pRF properties in the current study, are likely to be subtle and affect all age groups similarly.

It is also conceivable that some of the improvements in visual discrimination observed beyond age 6 years are driven by changes in non-visual processes such as more sustained attention, reduced response bias, or more adult-like setting of decision-bounds. Each of these processes could lead to underestimation of true performance, and be wrongly interpreted as visual development if unaccounted for (Manning et al., 2018; Leat et al., 2009). Neuroimaging measures are less sensitive to these confounds because tasks are typically orthogonal to measures of visual sensitivity. Nevertheless, non-visual development is unlikely to account for all improvements in visuospatial performance in late childhood, because different spatial abilities develop at different rates, even when task demands are very closely matched (Braddick and Atkinson, 2011; Kiorpes, 2015; Skoczenski and Norcia, 2002).

Thus, whilst more work is needed to understand the factors driving visual development in childhood, the current study provides the significant new insight that prolonged pRF shape, size, and position tuning in early visual cortex is unlikely to play a major role in this process. Moreover, the in-depth characterisation of pRF properties across the child visual field presented here, will form an important foundation for future studies on visual plasticity in healthy and visually impaired populations - both of which are increasingly important areas of research given recent developments in gene- and stem-cell treatments of paediatric eye disease. One direct application for future work, for example, is that retinotopic templates based on adult-brains (Benson et al., 2012) may be used to investigate retinotopic connectivity in primary-school aged children.

In conclusion, we report that population tuning in early visual cortex remains largely consistent between ages 6–12 years, and adulthood. This suggests that the development leading to improvements in basic visuospatial abilities does not entail substantial changes in cortical acuity as reflected in pRF size or organisation. Instead, improved perception may be due to more complex tuning properties, or to higher-level processes that facilitate more optimal use of spatial information in the early visual system. This may include more efficient information read-out and top-down modulation, or sustained attention and decision-making. Our findings highlight the importance of understanding visual development at a neural level, to disentangle the processes that drive behavioural improvements, and provide an important developmental baseline against which clinical populations can be compared.
