# Supplementary material for: Population receptive field tuning properties of visual cortex during childhood
Source: Dev Cogn Neurosci. 2019 Jan 8;37:100614. doi: 10.1016/j.dcn.2019.01.001 (PMC6969313; doi:10.1016/j.dcn.2019.01.001)
Supplement: Supplementary file 1 [file mmc1.docx]

**Supplementary Figure 1**

**
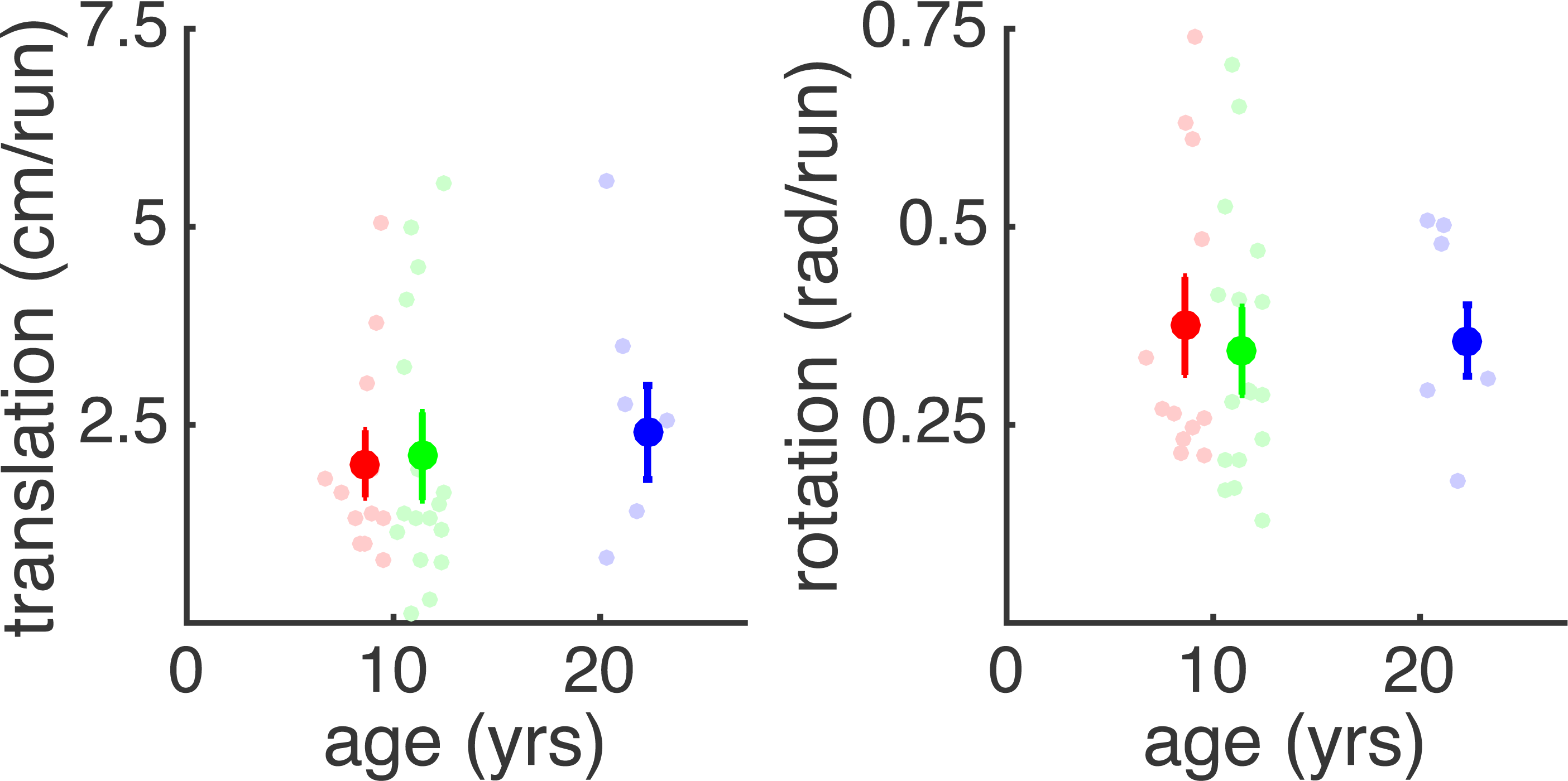
**

To minimize age-related confounds due to head-movements in the scanner, we removed participants who made excessive sharp head-movements that are difficult to correct for using realignment (Diedrichsen and Shadmehr, 2005) from the dataset. To quantify head-movements, we computed the *absolute* rotation (mean roll, pitch and yaw in radians) and *absolute* translation (translations along the x-, y-, z- hypotenuse in mm) from each scan to the next. Participants who exceeded 1mm translation or 3° rotation from one scan to the next in more than 3 volumes in the 4 runs were excluded. To quantify head movement in the remaining participants, we summed all scan-to-scan displacements across the entire run. In Figure 1_1, mean rotations and translations are plotted per individual (lighter data points) and per age group. Error bars are bootstrapped 95% confidence intervals. ANOVA’s revealed no significant age differences in translation (F(2,36)=0.131, *p*=0.88) or rotation (F(2,36)=0.086, *p*=0.91). Thus, these head-movement exclusion criteria successfully distinguished between participants who moved a lot and who moved little, resulting in age groups well matched on this potential confound.

**Reference**

Diedrichsen J, Shadmehr R (2005) Detecting and adjusting for artifacts in fMRI time series data. NeuroImage 27:624–634.

**Supplementary Figure 2**

The difference of Gaussion pRF model described below was fit to the time courses of each surface vertex following the same procedures as described in the method.

$$e^{-\frac{{(X-x0)}^{2}+{(Y-y0)}^{2}}{{{2\sigma}_{1}}^{2}}}-{DoG}_{ratio}{*e}^{-\frac{{(X-x0)}^{2}+{(Y-y0)}^{2}}{{2\sigma_{2}}^{2}}}$$

Free parameters σ_1_, σ_2_, and DoG ratio were then averaged per group and eccentricity bin. Shaded errorbars indicate the bootstrapped 95% confidence interval. Bootstrapped ANOVA’s were run separately for each eccentricity, and tested for significance at a false discovery rate of 0.05 per parameter. In line with the overlap in errorbars across the data of the three age groups in Figure 2_1, this analysis revealed no significant age differences in any of the parameters: in Sigma1 (smallest uncorrected *p*=0.04), Sigma2 (smallest uncorrected *p* = 0.0058), and the DoG-ratio (smallest uncorrected *p* = 0.008).

**Supplementary Figure 3**

We computed visual field coverage based on all voxels in a given ROI with a good model fit (Rsq>0.1). Following procedures described in Grill-Spector et al. (2018), we first flipped the right hemisphere pRFs along the vertical meridian (y-axis), so that the visual field representations of the two hemispheres were in alignment. For each voxel, we then plotted the best-fitting Gaussian pRF (defined by x, y, sigma, and amplitude=1) across a 200x200 grid that represented a visual field of twice the size of the stimulus display (stimulus eccentricity =15°, projected visual field = 30°). At each pixel of this grid we identified the maximum across all pRFs in the ROI. To reduce contributions of noise, we bootstrapped the maximum pRF value 100 times across all pixel locations and took the average to generate individual visual field coverage maps. Age group maps for each ROI were then computed by bootstrapping across the individual average maps of each age group (Supplementary Figure 3A).

To compare visual field coverage statistically, we computed the average amplitude (set to maximally 1) within circular eccentricity bins (Supplementary Figure 3B). The borders of these bins increased along the visual field in steps of 1.5° from the centre coordinate of the screen up to 2x the maximal size of the stimulus. The resulting mean visual field coverage across the visual field, is plotted in Supplementary Figure 3B. Shaded errorbars indicate bootstrapped 95%CI. This analysis reveals a trend towards narrower coverage of the visual field at younger ages, especially in V3A, but the overlapping errorbars indicate that these differences were not significant.

**Supplementary Table 1**

|  | V1 | V2 | V3 | V3A | V4 |
| --- | --- | --- | --- | --- | --- |
| 95%CI  Lower Bound | -0.29 | -0.20 | -0.20 | -0.20 | -0.14 |
| 95% CI  Upper Bound | 0.16 | 0.19 | 0.15 | 0.27 | 0.29 |

Supplementary Table 1. Proportional confidence intervals on bootstrapped t-test of pRF size difference between 6 to 8-year-olds and adults

To investigate the limits of the (non-significant) age-difference in pRF size given the current data, we bootstrapped 95% confidence intervals on the difference in pRF size between 6 to 8-year-olds and adults for each ROI. The resulting CIs in Supplementary Table 1 are expressed as a proportion of the adult pRF size in the corresponding ROI. Following this maximum bound criterion, the plausible range for an in- or decrease in pRF size between ages 6-8 years and adulthood given current data, falls between 0-30% for all ROIs. Thus, while age differences in pRF size may be revealed with a larger sample size, these are likely to be small compared to individual differences reported in other studies (e.g., a 2-fold (200%) change in pRF size linked to adult Vernier acuity reported by Song et al., 2015).
